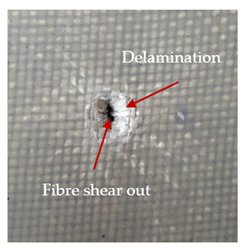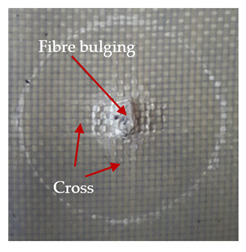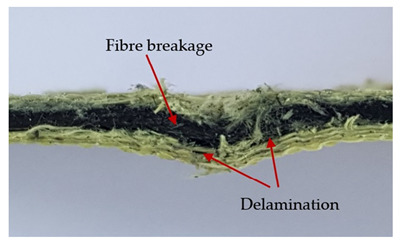# The Effects of Hybridisation of Composites Consisting of Aramid, Carbon, and Hemp Fibres in a Quasi-Static Penetration Test

**DOI:** 10.3390/ma13204686

**Published:** 2020-10-21

**Authors:** Joanna Pach, Natalia Frączek, Jacek Kaczmar

**Affiliations:** Department of Lightweight Element Engineering, Foundry and Automation, Faculty of Mechanical Engineering, Wroclaw University of Science and Technology, Wyb. Wyspiańskiego 27, 50-370 Wroclaw, Poland; natalia.fraczek996@gmail.com (N.F.); jacek.kaczmar@pwr.edu.pl (J.K.)

**Keywords:** hybrid composites, laminates, polymer-matrix composites, quasi-static penetration test, ballistic composites

## Abstract

The quasi-static penetration properties of hybrid laminates were experimentally investigated. Aramid fabrics, carbon fabrics, and short hemp fibres were applied as the reinforcements of hybrid and non-hybrid composite laminates with polyurethane–polyurea (PUR/PUA) matrix. The laminates were made by hand, in a mould. They were cured at room temperature for 24 h. Hybrid laminates consisted of aramid and carbon layers in two different configurations, i.e., aramid at the innermost layers and outermost layers. Aramid/PUR/PUA and carbon/PUR/PUA composites were fabricated for comparison purposes. Laminates were also prepared via an analogue sequence of laying the reinforcement layers with the addition of 5% by weight of hemp fibres in the PUR/PUA matrix. Quasi-static penetration tests (QSPT) were conducted using a tensile testing machine with a surface-hardened, hemispherical, steel punch (9 mm diameter tip), reflecting the geometry of the Parabellum projectile. A quasi-static puncture test was carried out until the laminate was perforated. The ratio between the support span (Ds) and the punch diameter (Dp) was SPR = Ds/Dp = 5.0. The results showed the influences of laminate hybridisation on the values of absorbed energy, punch shear strength, and damage mechanism in the QSPT test. The addition of hemp fibres to aramid laminates resulted in a positive hybridisation effect. The order of layers of aramid and carbon fabrics in hybrid laminates influenced the results obtained in the QSPT test.

## 1. Introduction

Polymer composites are of interest to researchers and engineers due to their potential applications in the automotive, aircraft, and defence industries. This has been shown by numerous scientific papers in which the results of quasi-static penetration and ballistic tests of polymer composites are presented [1,2]. Polymer composites are used in engineering due to their light weights, strength, stiffness, and specific strength (understood as the relation of the mechanical strength and the specific gravity). Fibre-reinforced polymer matrix composites for ballistic impact applications are classified into two main categories [1,3]: (a) high-strength/high-stiffness composites and (b) high-ductility/high toughness composites. Composite materials of the first type (a) are usually based on carbon fibre reinforced polymers. These materials are very effective in deforming or fragmenting a projectile, but they have limited ability to absorb the kinetic energy of the projectile. Composites of the second type (b) are characterised by the ability to absorb most of the kinetic energy carried by the projectile. In this role, composites reinforced with aramid fibres are the most used.

Aramid fibres are characterised by relatively low density, one of the highest specific modules, and high elongation at break. Aramid fibres and composites with the strengthening aramid fibres are used in various armour systems where high-speed energy absorption is required.

Polymer composites with aramid [4,5], carbon [6,7], or glass fibres [8,9] have been subjected to quasi-static and/or ballistic tests and are documented by numerous publications.

The quasi-static penetration tests were conducted in order to study the damage evolution and penetration resistance behaviour of the polymer laminates. They were also used to evaluate the effect of hybridisation on the energy absorption of laminated composites under transverse loading without a dynamic effect.

Increasingly, researchers’ attention is also focused on hybrid composites in which more than one type of reinforcement is used. The possibility of combining the properties of different types of reinforcements into one composite seems promising, but the process of forming hybrid composites may cause technological difficulties due to the different adhesion properties of the reinforcement to the polymer matrix.

The application of various reinforcing fibres as laminate layers (e.g., layers of glass and carbon fibres) increases the susceptibility to delamination between such different layers [10,11]. However, the replacement of carbon fibres in the surface layer with glass fibres does not necessarily lead to a decrease in impact resistance [12].

Experimental studies of hybrid composites were carried out by Bulut et al. [13]. Hybrid composites reinforced with carbon, aramid, and S-glass fibres were subjected to quasi-static puncture tests. The experiment was carried out for two coefficients: SPR = 2 and 5. As a result of the research, the authors found that with the increase of the SPR coefficient, the values of the penetration force, the shear stress, and the absorbed energy were higher. The authors found that the fibres placed in the outer layers of the laminate play important roles in the dissipation of energy and the damage mechanism of the structure.

The effects of hybridisation on the ballistic impact behaviours of hybrid composites were investigated by Bandaru et al. [14]. One of the aims of the work was to assess the ballistic performance of hybrid laminates with different hybridised stacking sequences. Among others, four-layer laminates in carbon, aramid, and glass fibres were tested, which were hybridized by introducing fabrics of other fibres in different orders in the laminate. The authors noted that placing the aramid fibres layer on the back side in carbon or glass laminates offers good ballistic impact resistance. It was found that among the tested laminates, the maximum ballistic limit was reached for aramid laminates hybridized with carbon fabrics [14].

In addition to testing laminates reinforced with aramid, carbon, and/or glass fibres, there are scientific tests in which natural fibres are applied in hybrid laminates.

The advantages of natural fibres are as follows: simple availability, low density, and relatively low prices. Due to the good damping of mechanical vibrations and the absorption of relatively high energy values during the impact, these composites are used in the automotive industry. Composites strengthened with the natural fibres do not create sharp edges when cracking, and this is their additional advantage.

Research results on hybrid composites were presented by Yahaya et al. [1]. The researchers focused on analysing hybrid composite damage after quasi-static penetration and ballistics tests. The tests were carried out on composites reinforced with layers of aramid fabric and kenaf fabric. The authors showed that in a quasi-static test the hybridisation effect positively affected the value of absorbed energy. However, in the case of ballistic tests, a lower ballistic limit of hybrid composites was noted compared to a non-hybrid composite reinforced with aramid fabric.

Positive effects of hybridisation in a quasi-static penetration tests of kenaf/aramid-reinforced polyvinyl butyral hybrid laminates have also been shown in another work [15]. Positive results have been obtained regarding increased energy absorption capacities of composites.

In composites in which good impact properties are required, continuous fibres are almost always applied as reinforcements. Short or long fibres in the form of mats are rarely used. The reason for the rare use of short fibres for materials with high impact resistance is the widespread belief that they are less effective in such applications. This is due to the lack of mechanical connection between the fibres and the structure of such reinforcement [11,16]. Cheeseman and Bogetti [12] indicated that the properties of mats do not decrease in terms of laminate resistance when shear stress and plug ejection dominate. It may be prudent to make the outer layers of ballistic laminate from a mat made of cheap glass fibres and the inner layers from materials that are more effective at absorbing energy but also more expensive [12]. An example of the use non-continuous fibres in ballistic composites is that of nanofibres [17].

Natural fibres in the forms of mats, rovings, and short cut fibres are used in the automotive industry as interior parts of vehicles [18]. The implementation of natural fibres in the polymer matrix may be aimed at reducing noise resulting from friction of plastic elements while driving. In addition, compared to glass fibres, natural fibres are safer for vehicle passengers during car accidents. Natural fibres do not form sharp edges that could injure a passenger as a result of damaged interior parts of the vehicle during a collision.

The search for new solutions leading to lighter, more impact-resistant, or cheaper laminates is still ongoing. This work is based on the analysis of hybrid composites, which consisted of assessing the impacts of hybridisation on the energy absorption capacities of composites. There are many studies of the behaviour of synthetic fibre composites subjected to quasi-static penetration tests. However, there are few reports on the impact responses of natural fibre/aramid/carbon hybrid laminate composites. The aim of the research was to design hybrid aramid/carbon/hemp composites and to evaluate their puncture resistance in a quasi-static penetration test.

## 2. Preparation of Samples and Methods of Their Evaluation

### 2.1. Materials and Impact Specimen Configurations

**Figure 1:**
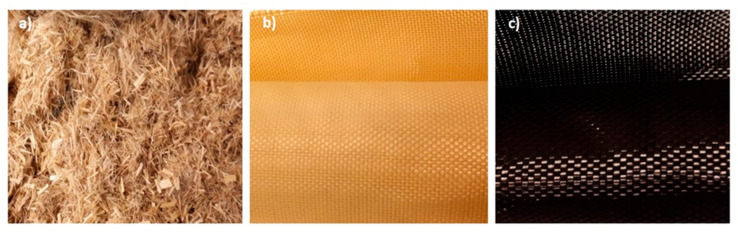
Types and forms of fibres used in laminates: (**a**) short hemp fibres, (**b**) aramid fabric, (**c**) carbon fabric.

In this study, the tested specimens were laminates and hybrid laminates manufactured from carbon fabric, aramid fabric, and short hemp fibres. Materials used as reinforcements are shown in Figure 1. Their mechanical properties are given in Table 1.

Plain woven aramid fibre with an areal density of 173 g/m^2^ (Twaron, distributed by Havel Composites, Cieszyn, Poland), plain woven carbon fibre (Kordcarbon) with an areal density of 200 g/m^2^, and hemp fibres (Cannabis sativa) were used as reinforcement fibres in the laminates. The hemp fibres used in this study were delivered by the Institute of Natural Fibres—(Poznań, Poland), and were cut in order to achieve a mean length of 4 mm. The fibres were dried at the temperature of 105 °C for two hours in a hot air cabinet. For alkali treatment, the pre-dried hemp fibres were soaked in an aqueous solution of sodium hydroxide (8 wt% NaOH) for two hours at room temperature (20 °C). Afterwards, the fibres were rinsed with distilled water until the filtrate reached pH 7. In succession, the fibres were dried again at 105 °C until a constant weight was reached. Modified hemp fibres were used because they are known to have better properties than unmodified ones, as described in detail in another study [18]. The resin used in this study was polyurethane–polyurea liquid (Almacoat Floor SL, Alma-Color, Gniew, Poland) with a density of 1.10 g/cm^3^. The resin was cured using joint cycloaliphatic amine, with a density of 1.05 g/cm^3^. The polyurethane–polyurea resin in a stoichiometric ratio of 100:13 was used in the common matrix.

**Figure 2:**
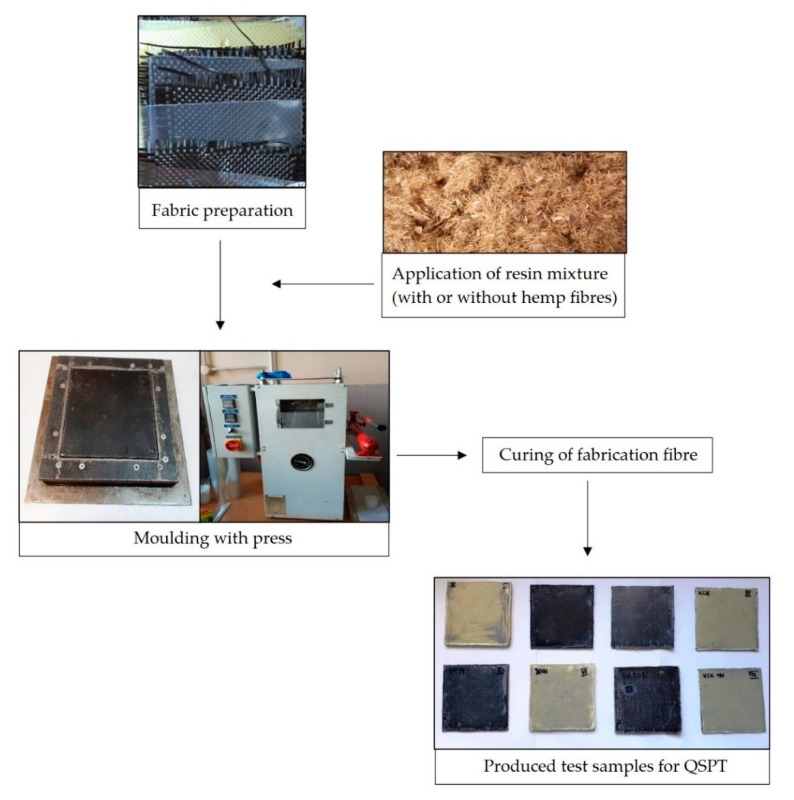
Production process of laminates.

Laminated fabrics with dimensions of 100 × 100 mm were prepared by hand via lay-up method and subjected to a pressure of 5 MPa in flat moulds under a press. Composites were cured by applying compression pressure at room temperature for 24 h. The lamination production process is shown in Figure 2.

**Figure 3:**
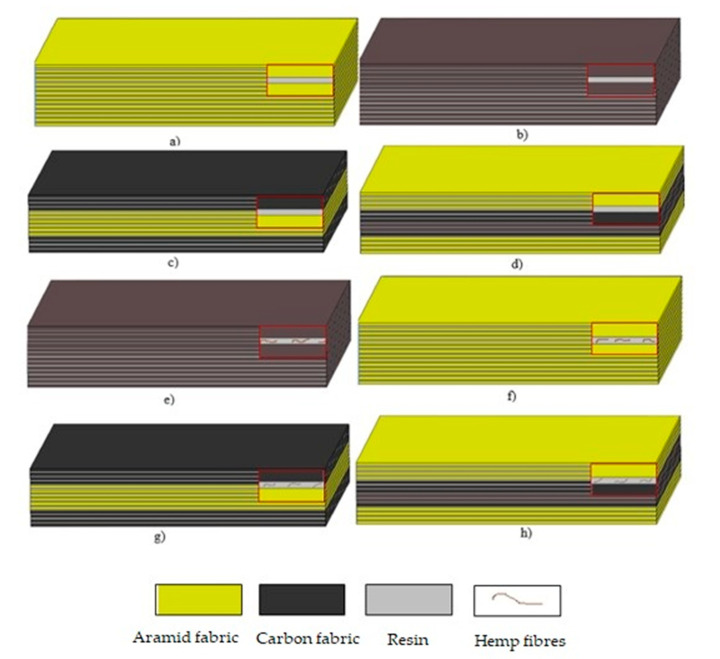
Non-hybrid (**a**,**b**) and hybrid (**c**–**h**) configurations of composite laminates.

The sequences of the fabrics in the laminates and the fibre volume fractions are given in Table 2. Laminates with aramid and carbon reinforcement were prepared. In the hybrid laminates, aramid fabric was placed in two different locations: the outermost or innermost layer. Laminates with an analogous sequence of laying the fabric layers saturated with a polyurethane–polyurea resin with 5% (by weight) short hemp fibres were also prepared. The configurations of samples are shown in Figure 3.

### 2.2. Quasi-Static Penetration Tests (QSPT)

**Figure 4:**
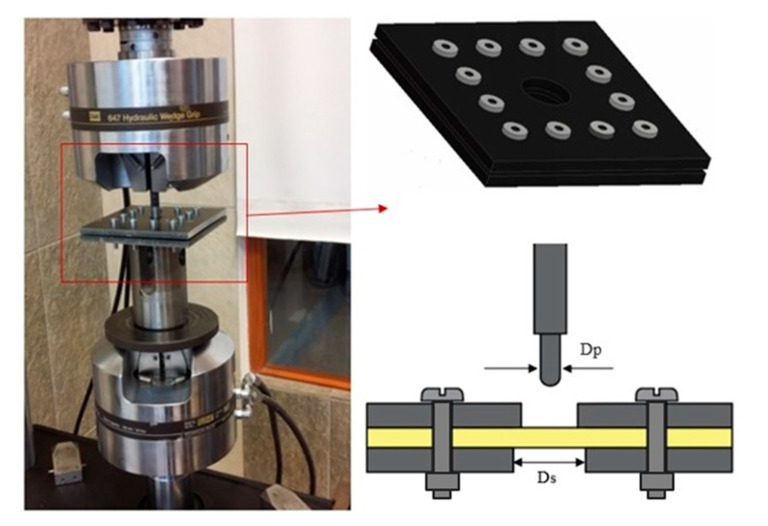
Schematic of a quasi-static punch shear test fixture.

A series of quasi-static punch shear tests were conducted using a surface-hardened steel indenter with a hemispherical, 9 mm diameter tip, reflecting the geometry of the Parabellum type projectile. The indenter was mounted at the MTS 810 tensile testing machine (Figure 4). The fixture consisted of a square cover and support plate (150 × 150 mm^2^, 8 mm thick) with a circular hole at the plate’s centre. A rectangular support was also used in addition to the support plate. The ratio between the support span and the punch diameter was SPR = Ds/Dp = 5.0. The laminate samples (square with a side of 100 mm) were fastened between the cover plate and the bottom support plate by 12 screws. Displacement control tests were performed at a crosshead displacement rate of 1.25 mm/s. The methodology of the quasi-static tests followed the standard ASTM D732 [19]. The purposes of the test were to show the progressive damage mechanism of composite laminates and to measure the relationship between the penetration force and the displacement. The force–displacement curves were reported. The mechanisms of damage formation in hybrid and non-hybrid laminates were analysed and compared. The tests were carried out until the last laminate layer was pierced and a plug was created around the indenter. The punch shear strength (PSS) was calculated Equation (1), which corresponds to the maximum force (Pmax) required to resist the plug from shearing out of the laminate [13].

(1) PSS = Pmax/(π Dp Hc)

where: Dp—diameter of the punch Hc—thickness of the composite.

**Figure 5:**
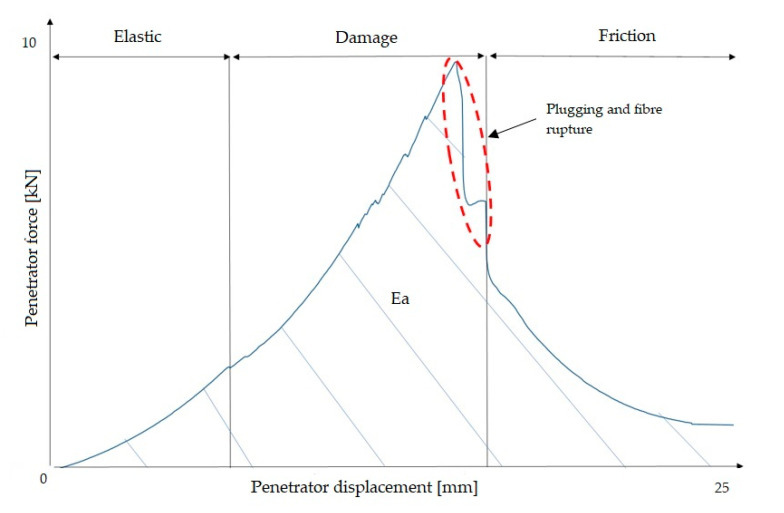
Diagram of laminate damage as a result of a quasi-static puncture test.

A diagram of the damage resulting from this test is shown in Figure 5. The mechanism of damage is divided into three stages: elastic, damage, and friction. In the first stage, the penetration force increases linearly and elastic deformations arise in the material. In the damage stage, the fibres are destroyed and the matrix is damaged, followed by the formation of a plug. Similar observations were presented in an article by Bulut et al., who conducted an experiment for laminates based on epoxy resin [13]. The last stage on the graph is the result of friction between the punch and the formed plug.

The quasi-static energy absorption (*Ea*) was quantified based on the area under the force–displacement curves, and it is the sum of the energy absorbed by the composite in a given area of damage, i.e., elastic energy, energy in the damage region, and frictional energy. The energy was determined by trapezoidal integration in a MatLab program.

## 3. Results and Discussion

### 3.1. QSPT Results

**Figure 6:**
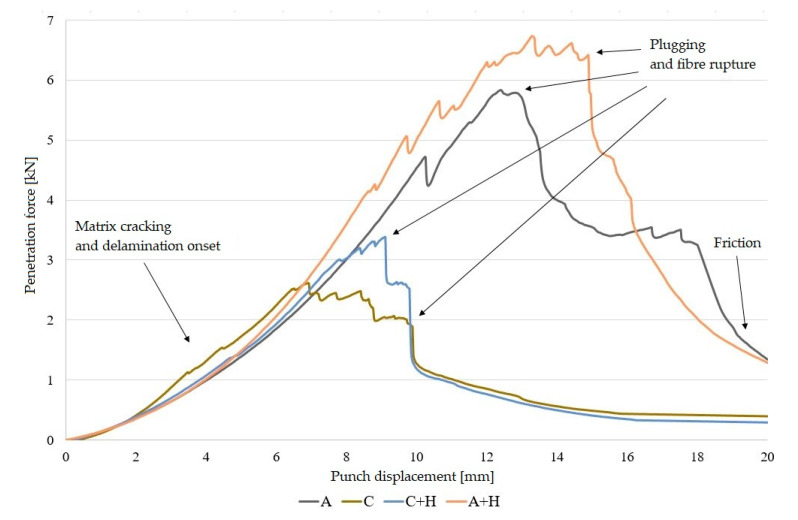
Variation of penetration force curves for non-hybrid laminates (designations as given in Table 2).

**Figure 7:**
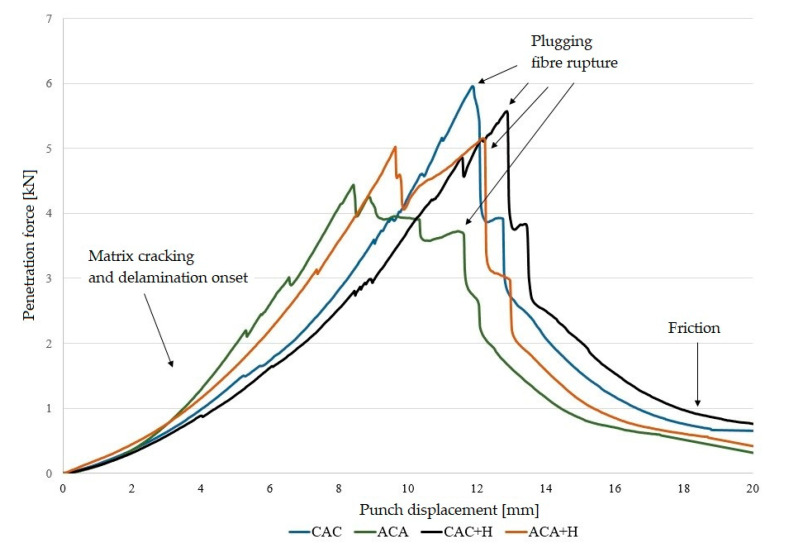
Variation of penetration force curves for hybrid laminates.

In order to compare the puncture resistance of polymer laminates, a quasi-static penetration test was carried out. The QSP test was carried out with one SPR because in this paper the authors focused on the structures and sequences of laminate layers rather than the effect of the SPR on the results of the QSPT. During the puncture test, penetration force–punch displacement curves were recorded. Figure 6 and Figure 7 present these curves for laminates and hybrid laminates based on an elastic matrix.

The mechanism of laminate damage presented in this paper is similar to those of epoxy-based laminates presented in other papers [1,8,13]. Authors A. Gama and J.W. Gillespie identified the QSPT damage mechanisms and sequences as: (a) transverse matrix damage, (b) initiation of punch shear at the loading face, (c) initiation of a shear plug formation, (d) initiation of interlaminar delamination, (e) completion of shear plug formation, (f) push-out of the shear plug through the penetration cavity, (g) large deformation of the rear half of the laminate, and (h) tensile fibre fracture and complete pushout of the plug and punch [8].

Similarly, in this research, an elastic stage was observed in each curve recorded in the puncture test, which was responsible for elastic deformations in the composites. The peaks on the curve indicate laminate matrix damage and the destruction of reinforcement fibres, until the formation of a plug in the bottom (rear) part of the laminate.

In non-hybrid laminates (A) and (C) and in laminates with the addition of hemp (A + H) and (C + H), the curves are similar, although the values of the total absorption energy are clearly higher for aramid and aramid composites with hemp fibres.

Differences were observed in the course of penetration curves for hybrid laminates. For hybrid laminates with aramid fibres in the outer layers, the curve is more horizontal in the area of damage. On the other hand, laminates in which the aramid fibres were placed in the inner layer exhibited the highest peak loads, resulting in a sudden load drop after plug formation. This was due to the different overall stiffness and ductile properties of aramid and carbon fibres.

**Figure 8:**
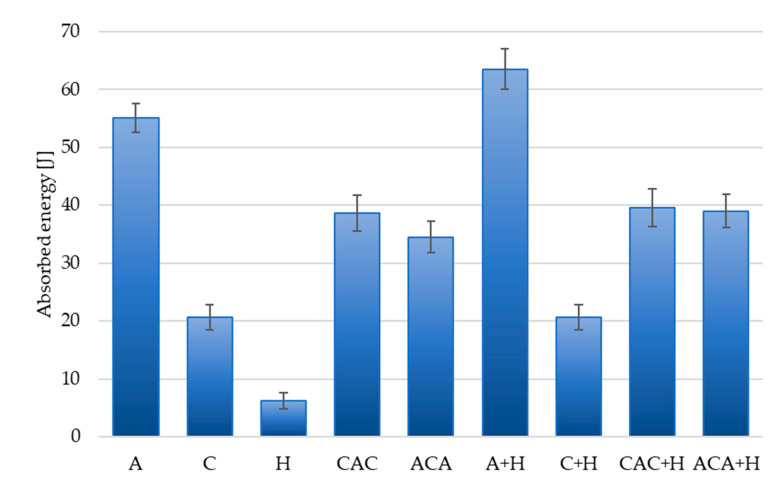
Total energy absorption of hybrid and non-hybrid composites.

**Figure 9:**
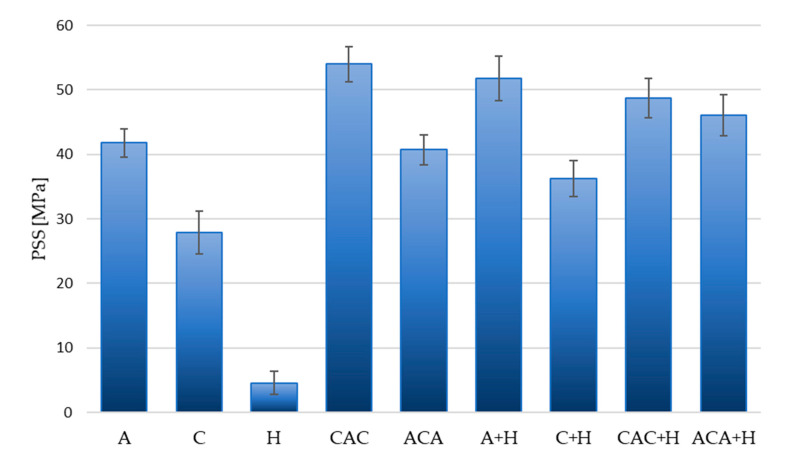
Punch shear stress distributions of laminates.

The results of calculations of absorbed energy (*Ea*) and punch shear strength (PSS) are presented in Table 3 and Figure 8 and Figure 9.

The highest Pmax value was recorded for laminates reinforced with aramid fabric (A) and hemp fibres (A + H), while the lowest values were for non-hybrid composites with short hemp fibres (H) and for laminates with carbon fibres (C). Higher values of Pmax for aramid laminates compared to short hemp fibres and carbon laminates were expected, as a result of the highest specific moduli and high elongation at break of aramid fibres. In hybrid laminates, higher Pmax and *Ea* values were recorded for laminates with carbon fibres as the outer layers of CAC laminates. It is worth emphasising that in these laminates there are fewer layers of aramid fabric than in ACA laminates. The disproportion between the number of layers of aramid and carbon reinforcements in hybrid composites was introduced intentionally, taking into account economic aspects.

The results obtained for laminates with hemp fibres are interesting. The introduction of short hemp fibres into the polymer matrix increased the total energy absorbed in the penetration test for laminates with aramid fabric (Figure 8) but did not significantly affect the value of absorbed energy of hybrid laminates.

It should be noted during the calculation and analysis of the results that the tested laminates differed in thickness, due to the character of the samples and the different thicknesses of the reinforcing fabrics. The analysis of *Ea* and Pmax only seems to be incomplete and can lead to erroneous conclusions. For a more complete interpretation of the results, the PSS (punch shear strength) was calculated while taking into account the thicknesess of the laminates tested (Figure 9).

The use of short hemp fibres in the polymer matrix improved the PSS for all laminates except for the CAC laminate. The increase in PSS value was 23% for laminate (A + H), 30% for laminate (C + H), and 13% for laminate (ACA + H). The PSS value for a laminate (CAC + H) compared to a laminate with an analogous reinforcement sequence without the addition of natural fibres was similar, but the moderately large dispersion of results should be taken into account here.

Our comparison of the PSS results indicates a significantly higher value for the CAC laminate compared to the ACA laminate, which is quite different from the results obtained for epoxy-based laminates with an analogous arrangement sequence of reinforcement layers [13]. The reasons for these differences can be explained in the application of another, more elastic polymer matrix, which also affects the differences in the mechanism of destruction. Higher values of Pmax, *Ea*, and PSS were achieved for CAC laminates. The impact resistances of composites are complex phenomena and the explanation of the results can be based on the theory of bending applied for the quasi-static puncture test. It can be assumed that during the first phase of the quasi-static puncture test, the compressive strength of the first layers of the laminate is of preliminary importance. Carbon fibres exhibit higher Young’s modulus than aramid fibres; therefore, their location in the first layers of the laminate seems to be more advantageous in the laminate. During the next phase of penetration of the punch into the laminate, the layers of fibres are sheared and stretched. In the last phase of puncture, after crossing the neutral layer, the fibre layers are stretched. High strength and elongation are the desired characteristics of the fibres used in puncture resistant laminates. Aramid fibres are more ductile than carbon fibres, but the elongation of the aramid fibres may induce additional destruction mechanisms, such as delamination. Taking into account the discussed properties of strengthening fibres, the choice of aramid fibres seems better than carbon ones. The most common mechanism of destruction in composites reinforced with relatively brittle carbon fibres is fibre cracking, while in composites reinforced with aramid fibres it is delamination. This is due to differences in the elastic strain values of the fibres. During impact loading, brittle fibres break faster than the warp, absorbing the impact energy. Aramid fibres are capable of large reversible deformations, and therefore delamination will occur before the energy is absorbed by the more fragile matrix.

The use of a more elastic matrix capable of large elastic deformations, as is the case for the tested laminates, may result in the absorption of part of the impact energy by elastic deformations of the composite without permanent damage [11].

**Figure 10:**
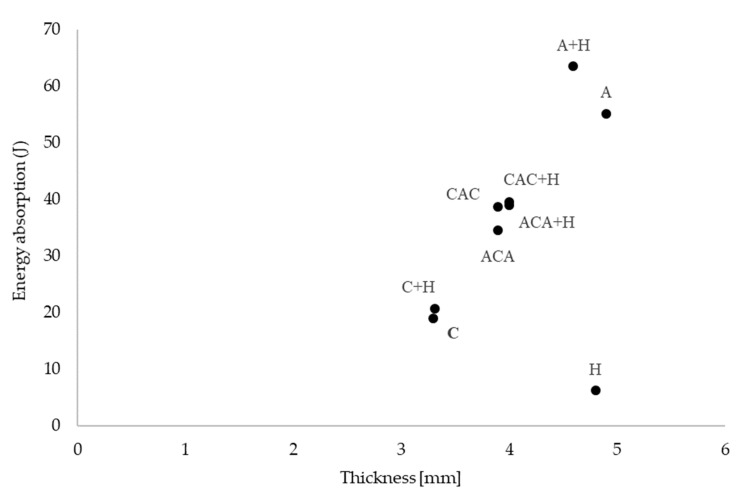
Effect of composite thickness on energy absorption.

The correlation between the energy absorbed during the puncture test and the thickness of the samples is shown in Figure 10.

The highest value of the total absorption energy was recorded for a hybrid laminate reinforced with aramid and hemp fibres (A + H), despite the lesser thickness compared to the aramid laminate (A). This confirms the beneficial effect of the addition of short hemp fibres on the values of absorbed energy.

In the case of laminates with the addition of carbon fibres (C and C + H), the laminate thicknesses were very similar and there were no differences in absorbed energy. For hybrid laminates with the addition of hemp fibres, a slight increase in the absorbed energy value was registered; however, no clear impact of the addition of hemp fibre on the increase of the absorbed energy value was observed.

### 3.2. Macroscopic Observations

The puncture effects of hybrid and non-hybrid composites in the QSPT test were characterised by taking pictures of the front and back sides of the samples, as shown in Table 4. QSPT was run until the laminate was punctured. In all investigated laminates there was breakage of fibres and formation of the plug, visible at the laminate side opposite to the acting force.

The energy absorption of the tested laminates is mainly associated with the deformation of the fibres up to their breakage. As a result of exceeding the endurance limit, among other things, stretching and shearing the fibres, and destroying the polymer matrix occur. There are also friction phenomena between the piercing element and the composite [20].

For aramid-based and hybrid laminates with aramid reinforcements in the outer layers of the laminate, a characteristic cross is visible on the front and/or back of each laminate. The tensile failures due to bending effects were dominant in the damage mechanisms.

The mechanism of cross formation in the place of stress concentration and cone formation in composites has already been discussed in the literature [11,20]. The fibres located directly under the penetrator are called primary yarns and can form a characteristic cross. These fibres are the first to be destroyed and carry higher loads than secondary fibres working in a smaller load range. Secondary fibres (yarns) cross with the primary fibres and are deformed as a result of mutual interaction. Stresses occurring in the secondary fibres depend on the distance from the penetrator, increasing in the vicinity of the penetrator [21,22,23].

The fibres can be destroyed as a result of exceeding the critical forces or they can be sheared [8,12,21]. The mechanism of fibre destruction depends on the fibre properties and impact speed. At higher speeds, shearing is more likely than fibre stretching, but this is also affected by the matrix type.

The laminates described in this paper were pierced at a constant speed. As can be seen from macroscopic observations, the effects of damage in the QSPT test largely depend on the types of fibres used. The carbon fibres used in the described laminates are far more brittle than aramid fibres. In the case of a laminate composed of aramid fibres, under the influence of puncture loads the fibres become more elastic and are stretched before breaking. A characteristic cross was formed in laminates in which aramid fibres were the last layers. In laminates in which the carbon fabric was the last layer, this effect was not visible, because they are more fragile and crack.

In order to analyse the changes occurring in subsequent layers of the laminate during puncture, cross-sectional images of laminates were taken. In the first layers of the laminate, compression of the fibres together with the polymer matrix was observed directly under the edge of the penetrator. As the penetrator sinks, a cone forms in the subsequent layers of the laminate, which indicates the stretching of the fibres. However, there are differences in the behaviour of aramid and carbon fibres. It was noticed that the cone on the back side of the laminate is larger for laminates containing aramid fibres rather than only carbon ones, which is explained by the greater ability of the aramid fibres to deform elastically.

Cross-sectional analysis of laminates also provides information on other types of polymer matrix damage. The type of damage that dominates during destruction depends on the impact parameters and the properties of the composite—both reinforcing fibres, warp, and interactions between them.

In this work an elastic matrix was used. The macroscopic images show little damage in the form of delamination. This may be due to the fact that the elastic matrix probably has greater resistance to damage initiation and propagation compared to the brittle matrix [24]. Thus, during the impact, some of the energy can be absorbed by the reversible deformation of the laminates without permanent damage.

### 3.3. Effect of Hybridisation

The first scientific research on hybrid composites began several decades ago. The beginning of the issue is related to the invention of carbon fibres, the main disadvantage of which was its high price. In order to lower the price while taking advantage of the unique properties of carbon fibre, hybridisation has become a very active research area [25]. The hybridisation effect of polymer composites was calculated, among other authors [1,13,26]. Bulut et al. calculated the hybrid effect in polymer laminates as a positive or negative deviation of mechanical properties from the rule-of-mixtures behaviour [13]. The application of this definition is not straightforward. The rule of mixtures needs a specific composition parameter, but it is not always easy to determine experimentally. Examples of such parameters are the relative volume fractions of low-elongation fibres (LE) and high-elongation fibres (HE) [25].

In the present paper, the effectiveness of aramid-carbon-hemp laminate hybridisation was evaluated based on previous work carried out by Al-Kinani et al. and Yahaya et al. [1,26]. The effect of the addition of carbon and hemp fibres in aramid laminates was determined with reference to the absorbed energy and the maximum carried load with respect to the non-hybrid aramid laminate specimen.

**Figure 11:**
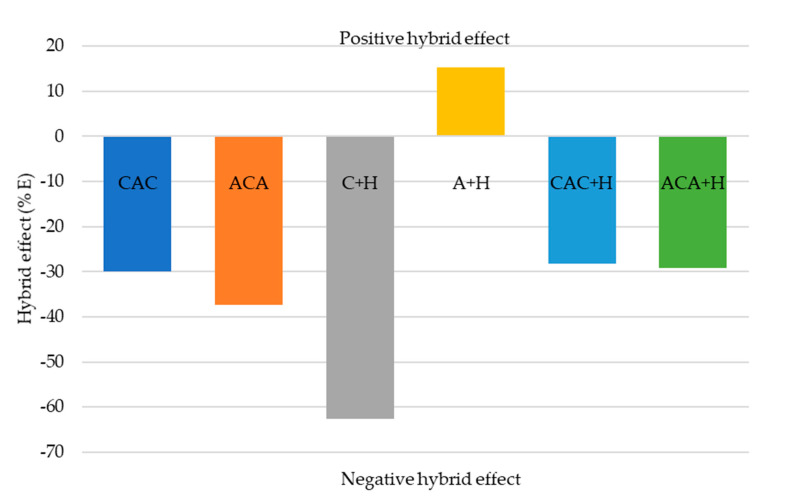
The percentage changes in absorbing total energy due to hybridisation.

**Figure 12:**
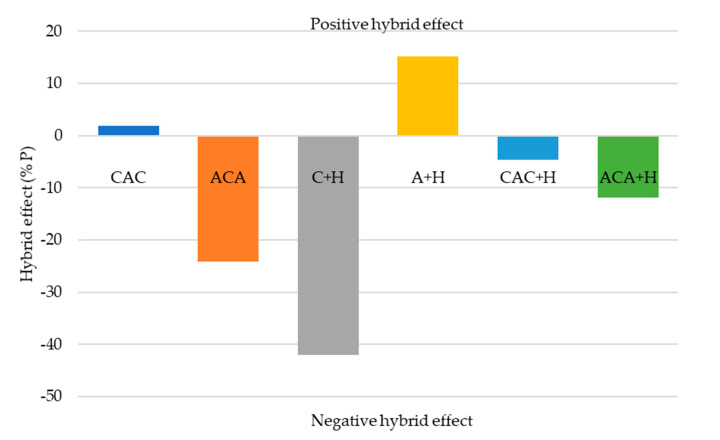
The percentage changes in maximum carried load (Pmax) due to hybridisation.

The resulting degree of hybrid effect: percentage changes in penetration energy (%*E*) and maximum load (%*P*) were calculated as follows:

(2) $$\%E=\left(Eh+Ea_{A}\right)/Ea_{A}\;\times 100\%$$

(3) $$\%P\;=\;\left(P_{h}+P_{A}\right)/P_{A}\times 100\%$$

where *Eh* and *Ea_A_*—absorbed energy of the hybrid and aramid laminates, respectively; and *P_h_* and *P_A_* are the maximum loads (Pmax) of the hybrid and aramid laminates, respectively. Hybridisation effect values of the samples are illustrated in Figure 11 and Figure 12.

The results showed that the A + H sample exhibited a positive hybrid effect. Low values for a laminate reinforced with carbon fabric with the addition of hemp fibres were expected because the reference points for calculations were for aramid laminate, which achieved higher values of absorption energy during breakthrough. The percentage changes in absorbing total energy due to hybridisation were not positive for composites of aramid and carbon fabrics. On the other hand, a positive effect of hybridisation on the maximum carried load was observed due to hybridisation for CAC hybrid laminate.

The authors of this work did not find any research in which the effect of hybridisation of composites composed of aramid and carbon fabrics and short natural fibres implemented in the polymer matrix was examined. As the test results show, the addition of short hemp fibres to the laminate matrix has a positive effect on the total absorbed energy and carried load. This effect may result from increased laminate stiffness.

The increase in the total impact energy of composites can occur, for example, as a result of introducing a hard mineral filler into the laminate polymer matrix [27]. An example of such a filler is sand. However, due to the high density of the filler, this solution was not used.

The effect of adding short, modified hemp fibres to a thermoplastic matrix was studied by Kaczmar and Pach [28]. It was found that the introduction of short hemp fibres into polypropylene increases tensile strength, Young’s modulus, and hardness, and decreases the relative elongation of composites, which is associated with the working reinforcement mechanism.

The implementation of hemp fibres to the laminates tested in this paper does not significantly increase the density, because the density of hemp fibres is similar to that of aramid fibres. However, the introduction of natural fibres into the polyurea/polyurethane matrix increases the rigidity of the system and increases the absorbed energy.

The hemp fibres used in the work underwent previous chemical modifications, which were aimed at removing a significant number of non-cellulosic substances and exposing the surfaces of the fibrils, which ensures better wetting by the liquid polymer during the production process of the composite material, and thus better adhesion at the interface between fibres and the polymer matrix is achieved.

## 4. Conclusions

The effects of hybridisation on the mechanical behaviour of aramid/carbon/hemp type of hybrid composites, consisting of short hemp fibres, aramid, and carbon fabrics, were studied. Aramid/carbon/hemp hybrid composites were prepared in different combinations. The current study focused on the effects of hybridisation and the effects of layering a sequence of aramid/carbon in the polyurethane–polyurea matrix. The damage and failure characteristics of composites were identified by punch shear-based tests using a flat-end punch for the support span (SPR 5). The essential findings of this work could be very useful concerning the conception of light-weight armour. The main conclusions from this study can be summarized as follows:

- Hybrid composites absorbed more penetration energy compared to carbon/polyurea–polyurethane composites and hemp/polyurea–polyurethane composites.
- The highest value of the total absorption energy was recorded for a hybrid laminate reinforced with aramid and hemp fibres (A + H). The implementation of hemp fibres into the matrix of hybrid laminates (ACA and CAC) did not result in a significant increase in the value of absorbed energy.
- The PSS value increased after the implementation of hemp fibres into the polyurea–polyurethane matrix in laminates with aramid (A + H) reinforcement by 23%, carbon (C + H) by 30%, and hybrid (ACA + H) by 13%, compared to laminates with an analogous reinforcement configuration without the addition of hemp fibres.
- Hemp–aramid hybridisation produced a positive effect in terms of energy absorption and maximum load changes compared to aramid/polyurea–polyurethane composites.
- The fibre arrangement sequence had an effect on the value of energy absorbed in the quasi-static penetration test. Hybrid laminates (CAC) with external carbon fibre layers absorbed more energy compared to ACA laminates. Similarly, for these laminates (CAC), higher punch shear stress values were also recorded.

## Figures and Tables

**Table 1:** Physical and mechanical properties of fibres.

Fibres	Density [g/cm^3^]	Tensile Strength [MPa]	Young’s Modulus [GPa]	Areal Density [g/m^2^]
Hemp	1.48	300–800	10–70	-
Aramid	1.44	2750–3000	82–124	173
Carbon	1.75–1.91	2500–3000	200–700	200

**Table 2:** Sequences of fabric arrangement and fibre volume fractions of the laminates.

Sample *	Stacking Sequence and Fibre Configuration	V_H_ (%)	V_A_ (%)	V_C_ (%)	Density [kg/m^3^]
A	(0⁰_A_/90⁰_A_)_7_	0.00	75.57	0.00	1276.12
C	(0⁰_C_/90⁰_C_)_7_	0.00	0.00	73.10	1370.21
CAC	(0⁰_C_/90⁰_C_)_2/_(0⁰_A_/90⁰_A_)_3_/(0⁰_C_/90⁰_C_)_2_	0.00	33.90	41.18	1262.14
ACA	(0⁰_A_/90⁰_A_)_2_/(0⁰_C_/90⁰_C_)_3_/(0⁰_A_/90⁰_A_)_2_	0.00	42.90	35.18	1126.54
C + H	[(0⁰_C_/90⁰_C_)_7_]_H_	2.94	0.00	72.03	1374.16
A + H	[(0⁰_A_/90⁰_A_)_7_]_H_	2.94	73.92	0.00	1255.80
CAC + H	[(0⁰_C_/90⁰_C_)_2_/(0⁰_A_/90⁰_A_)_3_/(0⁰_C_/90⁰_C_)_2_]_H_	2.94	31.37	42.13	1330.60
ACA + H	[(0⁰_A_/90⁰_A_)_2_/(0⁰_C_/90⁰_C_)_3_/(0⁰_A_/90⁰_A_)_2_]_H_	2.94	42.64	32.48	1200.97

* A—aramid; C—carbon; H—hemp fibres in PUR/PUA matrix.

**Table 3:** Comparison of QSPT results.

Sample	P_max_ (kN)	H_c_ (mm)	PSS (MPa)	*E_a_* (J)
A	5.84	4.94	41.78	55.14
C	2.61	3.31	27.88	19.00
H	0.62	4.80	4.56	6.24
CAC	5.95	3.90	53.99	38.67
ACA	4.43	3.85	40.69	34.53
A + H	6.73	4.60	51.76	63.54
C + H	3.39	3.31	36.19	21.00
CAC + H	5.57	4.04	48.76	39.54
ACA + H	5.15	3.96	46.02	39.01

**Table 4:** Front and back views of the samples after the penetration tests.

Sample	Front	Back	Cross-Sectional Views of the Samples after the Penetration Tests
A	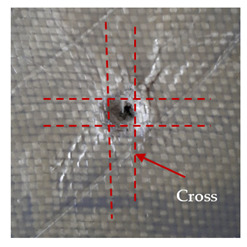	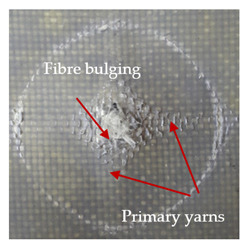	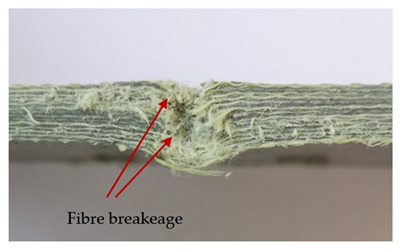
C	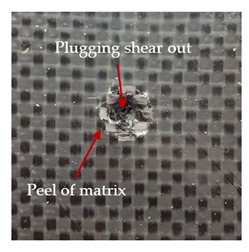	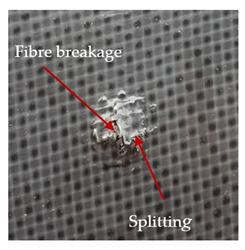	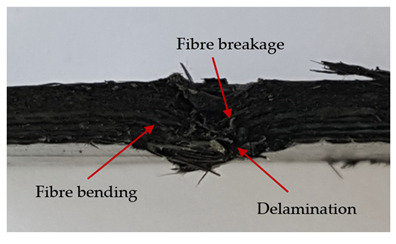
H	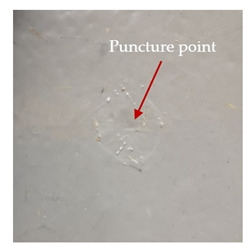	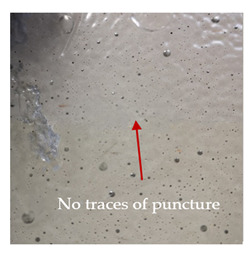	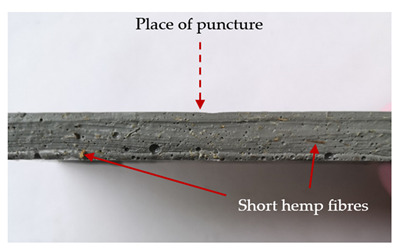
CAC	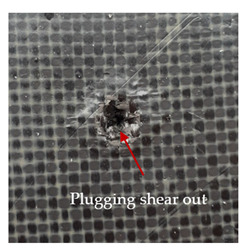	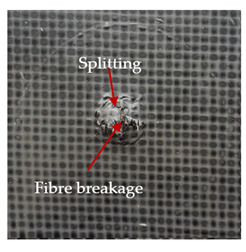	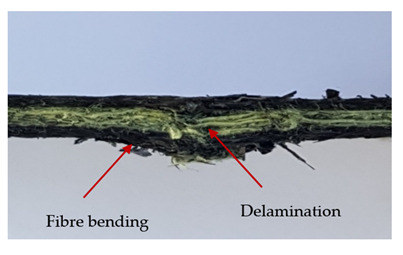
ACA	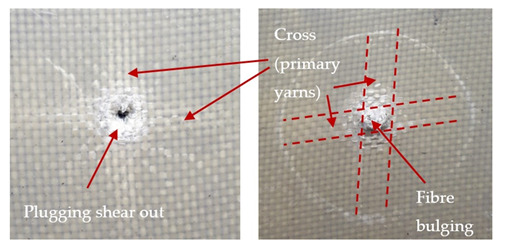		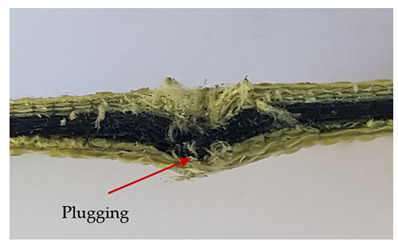
A + H	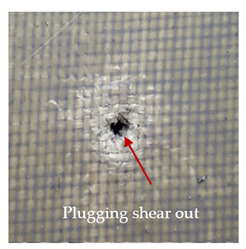	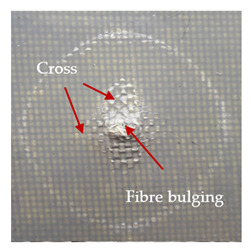	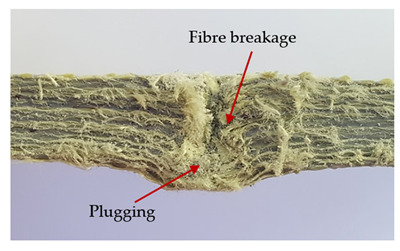
C + H	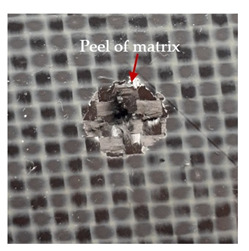	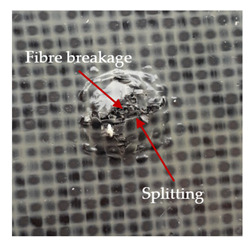	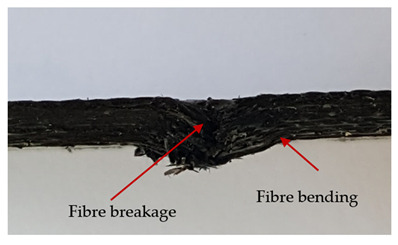
CAC + H	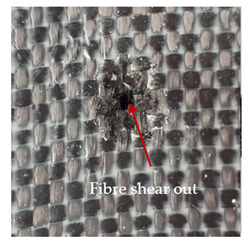	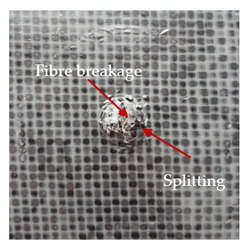	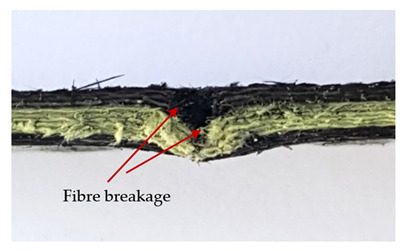
ACA + H